# Molecular characteristics and tumorigenicity of ascites‐derived tumor cells: mitochondrial oxidative phosphorylation as a novel therapy target in ovarian cancer

**DOI:** 10.1002/1878-0261.13028

**Published:** 2021-06-18

**Authors:** Yi Ding, Vera Labitzky, Karen Legler, Minyue Qi, Udo Schumacher, Barbara Schmalfeldt, Christine Stürken, Leticia Oliveira‐Ferrer

**Affiliations:** ^1^ Department of Gynecology University Medical Center Hamburg‐Eppendorf Germany; ^2^ Institute of Anatomy and Experimental Morphology University Medical Center Hamburg‐Eppendorf Germany; ^3^ Bioinformatic Core Facility University Medical Center Hamburg‐Eppendorf Germany

**Keywords:** ascites, chemoresistance, metastasis, ovarian cancer, OXPHOS, spheroids

## Abstract

Ovarian cancer disseminates primarily intraperitoneally. Detached tumor cell aggregates (spheroids) from the primary tumor are regarded as ‘metastatic units’ that exhibit a low sensitivity to classical chemotherapy, probably due to their unique molecular characteristics. We have analyzed the cellular composition of ascites from OvCa patients, using flow cytometry, and studied their behavior *in vitro* and *in vivo*. We conclude that ascites‐derived cultured cells from OvCa patients give rise to two subpopulations: adherent cells and non‐adherent cells. Here, we found that the AD population includes mainly CD90^+^ cells with highly proliferative rates *in vitro* but no tumorigenic potential *in vivo*, whereas the NAD population contains principally tumor cell spheroids (EpCAM^+^/CD24^+^) with low proliferative potential *in vitro*. Enriched tumor cell spheroids from the ascites of high‐grade serous OvCA patients, obtained using cell strainers, were highly tumorigenic *in vivo* and their metastatic spread pattern precisely resembled the tumor dissemination pattern found in the corresponding patients. Comparative transcriptome analyses from ascites‐derived tumor cell spheroids (*n* = 10) versus tumor samples from different metastatic sites (*n* = 30) revealed upregulation of genes involved in chemoresistance (*TGM1*, HSPAs, MT1s), cell adhesion and cell‐barrier integrity (*PKP3*, CLDNs, *PPL*), and the oxidative phosphorylation process. Mitochondrial markers (mass and membrane potential) showed a reduced mitochondrial function in tumoroids from tumor tissue compared with ascites‐derived tumor spheroids in flow cytometry analysis. Interestingly, response to OXPHOS inhibition by metformin and IACS010759 in tumor spheroids correlated with the extent of mitochondrial membrane potential measured by fluorescence‐activated cell sorting. Our data contribute to a better understanding of the biology of ovarian cancer spheroids and identify the OXPHOS pathway as new potential treatment option in advanced ovarian cancer.

## Graphical abstract

In ovarian cancer, detached tumor cell aggregates (spheroids) are regarded as ‘metastatic units’ that exhibit unique molecular characteristics. Transcriptome analyses from ascites‐derived tumor cell spheroids versus tumor samples from different metastatic sites revealed upregulation of genes involved in several cellular processes, including the oxidative phosphorylation. Response to OXPHOS inhibition in tumor spheroids points to a potential role of the OXPHOS pathway as new treatment option in advanced ovarian cancer.

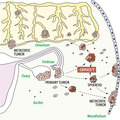

**Abbreviations**
ADs: adherent cells
ANGPT2: angiopoietin 2
APLNR: apelin receptor
ATP: adenosine triphosphate
B4GALT5: beta‐1,4‐galactosyltransferase 5
CCL: C‐C motif chemokine ligand
CD: cluster of differentiation
CHST4: carbohydrate sulfotransferase 4
CLDNs: claudins
DAB: 3,3'‐diaminobenzidine
DAPI: 4′,6‐diamidino‐2‐phenylindole
DNBseq: DNA Nanoballs sequencing
EGF: epidermal growth factor
EpCAM: epithelial cell adhesion molecule
FACS: fluorescence-activated cell sorting
FCS: fetal calf serum
FDR: false discovery rate
FFPE: formalin-fixed paraffin-embedded tissue
FIGO: International Federation of Gynecology and Obstetrics
FLG: filaggrin
HGSOC: high-grade serous ovarian cancer
HSPAs: heat shock protein family A
ICAM-1: intercellular adhesion molecule 1
ICC: immunocytochemistry
ITGA3: integrin subunit alpha 3
KEGG: kyoto encyclopedia of genes and genomes
KLF: kruppel-like factor
MARCO: macrophage receptor with collagenous structure
MMP: matrix metallopeptidase
MT1s: metallothioneins 1
NADH: nicotinamide adenine nucleotide
NADs: non-adherent cells
NEU1: neuraminidase 1
OvCa: ovarian cancer
OXPHOS: oxidative phosphorylation
PBS: phosphate-buffered saline solution
PCA: principal component analysis
PDGFRß: platelet-derived growth factor receptor beta
PKP3: plakophilin 3
PPL: periplakin
RBC: red blood cells
SCID: severe combined immunodeficiency
TAMs: tumor-associated macrophages
TGM1: transglutaminase 1
UKCCCR: United Kingdom Coordinating Committee of cancer Research

## 1 Introduction

Ovarian cancer represents the most common cause of death among women with gynecological malignancies, with an expected 5‐year mortality rate of nearly 60%. This low survival rate, that has modestly improved in the last decades, is mainly caused by often late disease detection. As ovarian cancer is asymptomatic at an early stage, around 70% of all diagnosed patients already show peritoneal metastasis at diagnosis [1]. Dissemination of ovarian cancer cells is mainly limited to the peritoneal cavity, where small tumor deposits can be detected frequently in the omentum and peritoneum. Unlike other solid cancers, ovarian cancer rarely disseminates through the bloodstream, although pelvic and para‐aortic lymph nodes are often involved. Frequently, patients develop ascites, a pathological fluid within the abdominal cavity containing tumor cells but also cells from no tumorigenic origin and diverse soluble factors that create a favorable environment for tumor growth and spreading. Tumor single cells and more frequently tumor cell aggregates or so‐called floating spheroids can be found in the ascites fluid [2, 3], the last representing the main source for peritoneal metastasis. Also, in advanced ovarian cancer patients without ascites, tumor cell spheroids are also present in the peritoneal lavage fluid collected during surgery. Recently, using an *in vivo* ovarian cancer model, it could be shown that multicellular detachment from the primary tumor rather than single tumor cell aggregation within the ascites fluid represents the main source of tumor spheroids. Here, tumor cell aggregates in ascites consist of neighboring cells from the primary tumor, and in turn, each single spheroid might develop an individual metastatic lesion [4]. These observations are in line with the genetic clonal mapping of ovarian cancer patients, showing that most metastatic sites from individual patients were phylogenetically pure [5].

Tumor cells within cell spheroids exhibit a survival benefit in comparison to single floating cells due to the protective microenvironment created by cellular interactions with other tumor and non‐tumoral cells. Recent *in vivo* data suggest an essential role of tumor‐associated macrophages (TAMs) in the formation of tumor spheroids and tumor progression. Here, TAMs within the spheroids secrete epidermal growth factor (*EGF*) and lead to upregulation of integrin and intercellular adhesion molecule 1 (*ICAM‐1*) in tumor cells [6]. Furthermore, tumor cells within the spheroids show an enhanced chemoresistance, mainly due to the lower incorporation and poor diffusion rates of chemotherapeutic drugs in such multicellular structures [7, 8]. Additionally, chemoresistance might be also caused, by the low proliferative and low metabolic cellular activity, which are typical characteristic of detached tumor cells and cell aggregates, as those floating in the peritoneal fluid or ascites [3].

In conclusion, ovarian carcinomas are highly heterogeneous tumors and tumor cell aggregates (spheroids) that detach from the primary tumor comprise unique clones, which might or might not survive in the peritoneal fluid. We assume that those spheroids that persist and are able subsequently to attach to the peritoneal cavity can be considered as ‘metastatic units’. These cellular structures share certain biological characteristics that might be useful in the development of new therapeutic strategies.

The aim of the present study is to characterize the cellular composition and the tumorigenic potential of the different subpopulations included in the ascites fluid of ovarian cancer patients. Further, a first insight into the specific molecular characteristics of tumorigenic ascites‐derived tumor cell spheroids was accomplished by RNA‐seq analyses.

## 2 Materials and methods

### 2.1 Patient material

Ascites was collected from patients diagnosed with advance ovarian cancer at the University Medical Centre Hamburg‐Eppendorf between 2017 and 2020. Ascites was obtained during debulking surgery from patients with primary and recurrent disease. Detailed patient characteristics are presented in Table S1. All patients gave written informed consent to access their biomaterial and review their clinical records according to our investigational review board and ethics committee guidelines (#190504 and PV6012) in the University Medical Centre Hamburg‐Eppendorf. Clinical parameters were retrieved from a detailed institutional database providing information on clinicopathological factors, surgical, and therapeutic procedures as previously described {Kuerti, 2017 #54}. This study is in compliance with the Declaration of Helsinki.

### 2.2 Preparation, cultivation of ascites‐derived cells, and isolation of ascites‐derived spheroids

Ascites or lavage from advanced ovarian cancer patients were centrifuged at 300 ***g*** for 5 min at room temperature. Supernatants were collected and frozen down at −20 °C for other purposes. Cell pellets were resuspended in RBC lysis buffer (Red Blood Cell lysis buffer; Invitrogen, San Diego, CA, USA) and incubated for 15 min at room temperature. After 5‐min centrifugation at 300 ***g***, the cell pellets were washed with PBS (Sigma‐Aldrich, St. Louis, MO, USA) and resuspended in PBS. 10 µL of cell suspension was mixed with 10 µL of a 0.04% trypan blue solution, and cell amount, cell size, and aggregation status were observed under the microscope.

Ascites‐derived cells were cultured in MCDB medium (MCDB 105 Medium and Medium 199 (1 : 1) supplemented with 10% fetal calf serum (FCS) and penicillin/streptomycin (2 mm; 1%; Thermo Fisher Scientific, Waltham, MA, USA) or in the clarified supernatant of the ascites at 37 °C on low‐attachment plates in the presence of 5% CO_2_ and 95% humidity. Here, some cells floated as spheroids or single cells in the medium (NADs) while some cells attached to low‐attachment plates (ADs).

For some experiments, large‐sized cell aggregates present in the ascites‐derived pellet were separated by using 15 µm cell strainers (pluriSelect; Leipzig, Germany). Briefly, the cell pellet was resuspended in PBS and put on the top of the cell strainer softly shanking until no fluid in the upper part was observed. Additional 5–10 mL PBS was added to the cell strainer to flush and wash the cells. The fluid containing all cells < 15 μm was collected in a 50 mL tube. Subsequently, the cell strainer was turned upside down and cells and cell aggregates retained in the strainer were flush back onto a new 50‐mL tube using 5–10 mL PBS.

### 2.3 *In vivo* intraperitoneal mouse model

Ascites‐derived cells (aprox. 3 × 10^6^ cells) from the original ascites cell pellet or from the separated tumor spheroid fraction of HGSOC patients were resuspended in MCDB medium without FCS (200 µL) and injected into the peritoneal cavity of immunodeficient female mice (CB17/Icr‐Prkdcscid/IcrIcoCrl (SCID; Charles River, Wilmington, MA, USA) or C.129S6(B6)‐Rag2tm1Fwa N12 (Rag2‐Model 601, Taconic; Hudson, USA), as previously described [9]. Due to the limited number of ascites‐derived cells available after preparation, habitually one mouse was injected per patient sample. For some patient samples, injections with cells corresponding to different size fractions were performed. In total, 30 mice were included in this study. The animals were housed with a 12‐h day–night cycle in a temperature‐ (21 °C) and humidity‐ (50%) controlled room. All mice were kept in individually ventilated cages under pathogen‐free conditions, fed with sterile standard food and water *ad* *libitum*. Mice that showed strong signs of tumor progression (ascites, shaggy coat, and loss of appetite; [10]) were anesthetized with xylazine/ketamine (120/16 mg/kg body weight, Bayer, Leverkusen, Germany/Graeub, Bern, Switzerland) and sacrificed after terminal cardiac blood collection by cervical dislocation. The dissemination pattern found was documented, tumors at the injection site, metastases, and lungs were excised, frozen or formalin‐fixed and embedded in paraffin. Animal experiments were conducted according to the UKCCCR guidelines for the welfare of animals in experimental neoplasia [10]. The mouse experiments were approved by the local licensing authority (Freie und Hansestadt Hamburg, Behörde für Gesundheit und Verbraucherschutz, Amt für Verbraucherschutz, project #G16/55).

### 2.4 Flow cytometry analysis

Cell pellets (ca. 500 000 cells) were washed with PBS, centrifuged (5 min at 1000 ***g*** and 4 °C), and resuspended in 100 μL antibody solution. All antibodies used were diluted in a PBS solution containing 1% BSA and 20% AB blocking solution (GRIFOLS; Barcelona, Spanien). Unstained samples were measured in the same blocking solution. The first antibody panel used to characterize the different cell population in the ascites‐derived cells included antibodies from BD Bioscience company against FITC‐CD45, APC‐CD90, BV421‐EpCAM (epithelial cell adhesion molecule), and PerCP‐Cy5.5‐CD24 as well as the Fixable Viability Stain 575v for cell viability assessment. For further characterization of the Ads, a new panel including APC‐CD90 (BD Bioscience, San Jose, CA, USA), APC/Cyanine7‐Podoplanin (BioLegend, San Diego, CA, USA), and PE‐mesothelin (R&D system, Minneapolis, MN, USA), and Pacific Orange™ succinimidyl ester (Thermo Fisher Scientific) was used. Further, we used the stem cell markers Alexa Fluor® 488‐CD44 (BioLegend) and APC‐CD133 (BioLegend) to analyze the NADs. All samples were incubated for 30 min at 4 °C in the dark. 500 μL PBS was directly added to each tube after incubation (centrifuging at 1000 ***g***/5 min/4 °C). Then, the pellets were resuspended with 500 μL 3.7% formalin in 0.1 m sodium phosphate buffer. For MitoTracker staining, MitoTracker green FM 20 nm and MitoTracker Red CMXRos 50 nm were incubated in basal MCDB105/M199 medium at 37 °C for 15 and 45 min, respectively. After washing with complete medium, the cell pellets were resuspended with complete medium and measured immediately. The samples were measured on the BD FACSCantoTM II Flow Cytometer. The evaluation was carried out with the FlowJo software version.

### 2.5 Fixation and embedding of ascites‐derived cells into agar

Ascites‐derived cells (*n* = 17) were resuspended in 5 mL of 3.7% formalin in 0.1 m sodium phosphate buffer and fixed at room temperature for 20 min. Then, fixed cells were washed twice with PBS. Then, cells were embedded in agar as previously described [11]. Briefly, the cell pellets were resuspended with 300 µL of 2% Difco™ Noble Agar (Becton, Dickinson, Sparks, MD, USA), which was preheated up to a temperature of 55 °C. After immediately centrifuged at maximum speed for 30 s, the cells in agar were cooled down on ice to form the solid agar piece. Subsequent paraffin embedding was performed using a Leica EG1160 Paraffin Embedding Center (Leica Biosystems; Nussloch, Germany).

### 2.6 Immunocytochemistry

Immunocytochemical analyses were performed as previously described [12]. Briefly, 4‐μm sections were cut from FFPE of ascites‐derived cells (*n* = 2), microwaved in citrate buffer pH6, and incubated overnight at 4 °C with the antibodies: EpCAM (Thermo Fisher Scientific, 1 : 600), CD45 (Dako, 1 : 50), and E‐Cadherin (Cell Signaling Technologies, Danvers, MA, USA, 1 : 500). Then the slides were incubated with a secondary antibody solution from Vector Laboratories: goat anti‐mouse or goat anti‐rabbit at room temperature for 30 min, respectively. For detection, slides were incubated with biotin‐labeled anti‐goat immunoglobulin (IgG), preformed ABC‐Complex (Vectastain, Vector Laboratories) and DAB‐substrate kit (Vectastain, Vector Laboratories). All slides were counterstained with hematoxylin. As negative controls, normal anti‐rabbit Immunoglobulin or anti‐mouse Immunoglobulin IgG1 (Dako Denmark A/S, Glostrup, Denmark) were used instead of primary antibody. Images were performed using an AxioVision40 Microscope (Carl Zeiss Imaging Solutions, Oberkochen, Germany).

### 2.7 Cytospin preparations

The cell viability was evaluated through staining with trypan blue. The highly viable spheroids enriched from the sample #7 was fixed as described before. 500 µL of 1 × 10^6^/mL cells suspension (1%BSA/PBS) was pipetted into the one‐funnel chamber mounted in Hettich Cyto‐Systems (Andreas Hettich; Tuttlingen, Germany). Centrifuge at 200 ***g*** for 3 min, then carefully remove the suspension. Let the slides dry at least 2 h at room temperature, wrap in foil and store it in −80 °C until use.

### 2.8 Fluorescence microscopy

For CD45, CD90, and EpCAM staining, the slides were blocked with 1%BSA/PBS at room temperature for 1 h. Then, antibody solution containing primary antibodies from BD Biosciences against FITC‐ CD45 (1 : 20), APC‐CD90 (1 : 125), and PE‐EpCAM (1 : 100) were added on the top of slides, incubated in 4 °C for 30 min, and washed three times with PBS. Antifade Mounting medium with DAPI (Vector Laboratories; San Francisco, USA) was added to the cover slide and then attached to the slide. The slides were further observed under the fluorescence microscope (Keyence BZ‐900). For the staining of MitoTracker, MitoTracker green FM 100 nm and MitoTracker Red CMXRos 20 nm were diluted in basal MCDB105/M199 medium, and incubate for 30 and 15 min, respectively. After washing with complete medium, the cell pellet was resuspended with 15 µL mounting medium, and added to the cover slide, then attached to the slide. The slides were further observed under the fluorescence microscope (Leica TCS SP8 X). For the mitochondrial marker, MitoTracker green FM (100 nm) and MitoTracker Red CMXRos (20 nm) were diluted in basal MCDB105/M199 medium and incubate for 30 and 15 min, respectively. After washing with complete medium, the cell pellet was resuspended with 15 µL mounting medium, and added to the cover slide, then attached to the slide. The fluorescent images were collected with a laser scanning confocal microscopy Leica TCS SP8 X and analyzed using the software las x Core (Leica Microsystems).

### 2.9 RNA sequencing

Spheroids from HGSOC patients were isolated from ascites‐derived pellets and analyzed using FACs as described in the corresponding sections. Spheroids showing a tumor cell content higher than 70% were further used for RNA isolation. 10um tumor tissue sections from HGSOC patients were cut from cryo‐tumor material and the hematoxylin and eosin (HE) staining was performed as described before. The tissue was tailored if necessary to obtain at least up to 70% tumor content. Approximately 10 sections were used for RNA isolation. Here, the RNeasy Kit (Qiagen; Hilden, Germany) was used following manufacturer’s instructions. RNA quantity and quality were measured using an Agilent bioanalyzer (Santa Clara, CA, USA). Sequencing was performed by BGI Genomics (Shenzhen, China) using the DNBseq™ Technology Platform in 2 × 100 bp paired‐end mode. On average, 24.2 m (minimum: 20.6 m; maximum: 26.1 m) read‐pairs were obtained per sample.

### 2.10 Sequence data analysis

Data analysis was performed in collaboration with the bioinformatics core facility of the University Medical Center Hamburg‐Eppendorf. Sequence reads were aligned to the human reference assembly (GRCh38.95) using STAR (v2.7.0.f) [STAR] and differential expression was assessed with DESeq2 [DESEQ]. Differentially expressed genes (Log2‐fold change > 1 and FDR < 0.1) were further analyzed for over‐represented REACTOME pathways [REACTOME], KEGG pathways [KEGG], and Gene Ontology terms [GENEONTOLOGY] using WebGestalt 2019 [WEBGESTALT] [13].

### 2.11 Viability assays with ascites‐derived tumor spheroids

Ascites‐derived spheroids from five different high‐grade serous ovarian cancer patients were enriched with 15 µm cell strainers as described before. Approximately 1 × 10^4^ counted cells were seeded in each well of a 96‐well plate resuspended in MCDB medium (90 µL/per well). Then, a total of 10 µL MCDB medium containing metformin (Merck KGaA; Darmstadt, Germany), cisplatin (Pharmacy, University clinic Hamburg‐Eppendorf, Germany), IACS010759 (Selleck Chemicals, #S8731), metformin + cisplatin or IACS 010759 + cisplatin were added to each well to reach following final concentrations: 0, 5 mm metformin, 0, 3.3, 33.3 µm cisplatin and 50 nm IACS 010759. Each treatment condition was plated in triplicates. After 48 h, 80 µL volume of spheroids from each well was mixed with equal amount of CellTiter‐Glo® Luminescent Cell Viability Assay Chemistry (Promega; Madison, Wisconsin, USA) in a white 96 Well Polystyrene Microplate (Greiner; Bio‐one, Kremsmünster, Austria) and shacked for 10 min. After 20 min further incubate at room temperature, the cell viability was measured using a luminescence reader (BioTek; Winooski, VT, USA).

### 2.12 Statistic

For the *in vitro* viability assays, spheroids were plated in triplicates. Statistical analyses were performed using the graphpad Prism (graphpad Software; Inc., CA, USA). Statistical significance was determined using unpaired two‐tailed Student’s *t*‐tests. The assumption of homogeneity of variance was tested using Levene's test of equality of variances (*P* > 0.05). Results are given as mean ± SD or SE. Probability values less than 0.05 were regarded as statistically significant.

## 3 Results

### 3.1 Characterization of cellular components in ascites from ovarian cancer patients

**Fig. 1:**
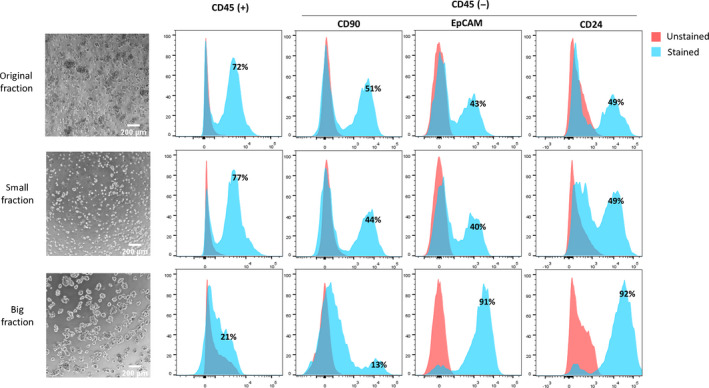
Representative analysis on ascites‐derived cells from one patient (#10). Pictures (left side) display the morphology of ascites‐derived cells in culture before (original pellet) and after separation into small and big fractions using a 15 μm strainer. Corresponding FACS results show the percentage of immune cells (CD45^+^), mesenchymal‐like cells (CD90^+^), and tumor cells (EpCAM^+^/CD24^+^) in each population. Stained samples (*n* = 37) were incubated with the corresponding antibody in a blocking solution (1%BSA, 20%AB blocking in PBS), and unstained samples were prepared in the blocking solution.

The ascites or lavage from OvCa patients (*n* = 141) were collected during debulking surgery. After the first centrifugation step, only samples containing a visible cell pellet were morphologically assessed by phase contrast microscopy immediately after collection on day 0 (*n* = 75). In 60% of the ascites samples, we found both single cells and cell aggregates (spheroids) as shown exemplary in Fig. 1 (original fraction), whereas the rest of the samples showed essentially a single‐cell population. To characterize the cellular components of ascites‐derived cells (*n* = 37), Fluorescence‐activated Cell Sorting (FACS) analysis was performed with an established antibody panel including CD45 (immune cells marker), CD24/EpCAM (tumor markers), and CD90 (mesothelial‐like cell marker) at day 0. These 37 samples measured by FACS included five low grade and 32 high‐grade ovarian cancer samples, different FIGO stages (IB: *n* = 1, IIIB: *n* = 5, IIIC: *n* = 2, and IV: *n* = 8) as well as four recurrent tumors. After excluding dead cells, a high heterogeneity regarding the content of CD45^+^, CD90^+^, CD24^+^, and EpCAM^+^ cells was observed among samples (*n* = 37). The tumor cell population, defined as EpCAM^+^ strongly varied from 0.8% to 99.8% among the ascites samples, whereas the range of immune and mesenchymal‐like cells was between 2.3% to 95.8%, respectively.

**Fig. 2:**
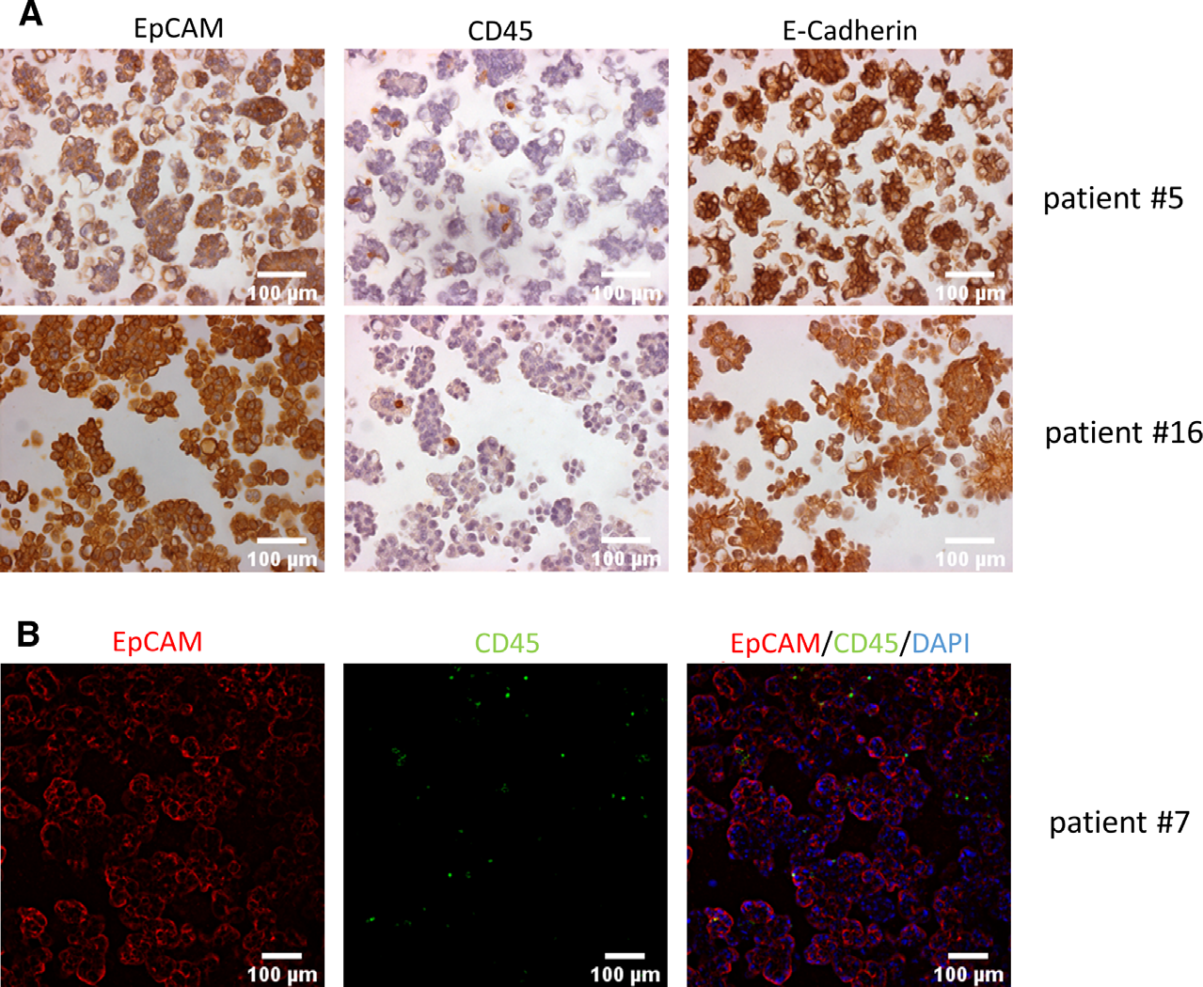
Immunocytochemistry and immunofluorescence analysis of spheroids from ovarian cancer patient ascites. Spheroid samples from 3 different OvCa patients were analyzed. (A) Paraffin‐embedded spheroids from two different patients (#5 and #16) showing strong EpCAM and E‐Cadherin expression and a few CD45^+^ cells. (B) IF staining of spheroids from patient #7 showing a similar pattern, namely a high percent of EpCAM^+^ and few CD45^+^ cells embedded in the aggregate structures.

Further, cell strainers of 15 µm were used to separate the original pellet into a small and a large cellular fraction. The multicellular aggregates (spheroids) were mainly collected in the large fraction, while the cell population smaller than 15 µm, defined as small fraction, included only single cells (Fig. 1). After this filtering step, the CD24^+^/EpCAM^+^ cell population was strongly enriched in the large fraction compared to the original and small pellet, while most CD45^+^ and CD90^+^ cells were found in the small fraction, which contained a relatively low amount of CD24^+^ and/or EpCAM^+^ single cells. Figure 1 displays representative FACs analysis showing a clear reduction of immune (CD45^+^: from 72% to 21%) and mesenchymal‐like cells (CD45^−^/CD90^+^: from 51% to 13%) in the large fraction and a remarkably increase of ovarian cancer cells (CD45^−^/EpCAM^+^ and CD45^−^/CD24^+^: 43% to 91% and 49% to 92%, respectively). The purity of the cell spheroids enriched in the large fraction could be corroborated by ICC and IF analysis. Figure 2 displays exemplary pictures from three samples showing cell aggregates with strong cellular EpCAM and a few immune cells (CD45^+^). Additionally, a strong E‐Cadherin staining was found in two samples by ICC. In line with the FACs analysis, sample #7 showed in the IF a high content of EpCAM‐positive cells and a lack of CD90‐positive cells.

### 3.2 Cellular characterization of different subpopulations arising from ascites‐derived cells *in vitro*

**Fig. 3:**
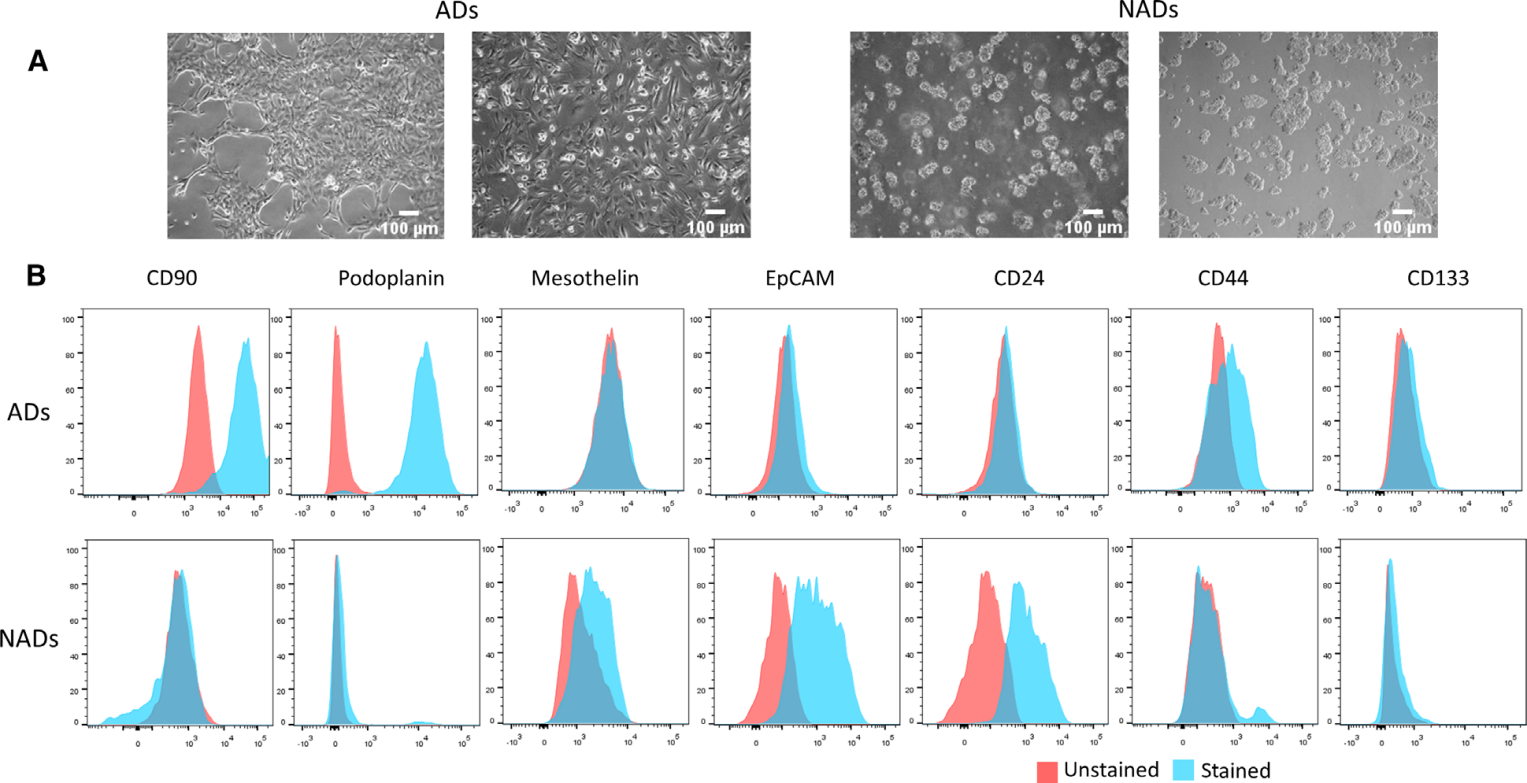
*In vitro* behavior of ascites‐derived cells. Ascites‐derived cells from 10 OvCa patients were analyzed. (A) Representative pictures of ascites‐derived cells from two ovarian cancer patients after 5 days *in vitro* culture. Two different cell populations can be observed, adherent elongated spindle‐shaped mesenchymal‐like or cobblestone‐shaped epithelial‐like cells (ADs) and multicellular aggregates (spheroids) and small single cells floating in the supernatant without attachment (NADs). (B) Representative FACs analysis of ADs and corresponding NADs from sample #15 showing strong CD90/Podoplanin/CD44 expression and EpCAM and CD24 expression, respectively. Stained samples were incubated with the corresponding antibody in a blocking solution (1%BSA, 20%ABblocking in PBS), and unstained samples were prepared in the blocking solution.

Ascites‐derived cells (day 0) were cultured in low‐attachment plates with MCDB medium for up to 2 weeks. Here, two different cell populations could be distinguished after approx. 5 days cultivation time as previously described by Latifi *et al*.[2]: adherent elongated spindle‐shaped mesenchymal‐like or cobblestone‐shaped epithelial‐like cells (ADs) and multicellular aggregates (spheroids) and small single cells floating in the supernatant without attachment (NADs; Fig. 3A). Both cell populations showed a remarkably different proliferative behavior. ADs were typically highly proliferative, whereas the NAD population remained quiescent but viable for up to 2 weeks cultivation time. In order to better characterize these two cell populations, an additional FACs panel including a tumor marker (EpCAM), stem cell markers (CD44, CD24, and CD133) and mesenchymal‐like and mesothelial cell markers (CD90, podoplanin and mesothelin) was established. Thus, the AD population (*n* = 9) exhibited a high content of CD90^+^, podoplanin^+^ and to some extent mesothelin^+^ and CD44^+^ cells, whereas NADs (*n* = 9) were mainly EpCAM^+^ and CD24^+^ (Fig. 3B and Fig. S1). Figure 3B displays representative FACs results from sample #15 showing a strong CD90, podoplanin, and CD44 positivity for AD cells, whereas the corresponding NAD cell population only express EpCAM and CD24. Additional FACS analyses from six AD/NAD pairs are shown in the Fig. S1.

Remarkably, 2 samples showed a different behavior *in vitro* (Fig. S2). They both contained low proliferative AD cells with an additional strong EpCAM, CD24, CD133 staining, suggesting a cancer stem cell phenotype.

These findings led us to the conclusion that in most of the ascites‐derived samples the highly proliferative population of ADs does not comprise ovarian cancer cells but rather a mesenchymal‐like cell population and cells from mesothelial origin. To prove this assumption, the tumorigenic potential of these cells was further tested *in vivo* using an intraperitoneal mouse model.

### 3.3 *In vivo* tumorigenicity of ascites‐derived cells

**Fig. 4:**
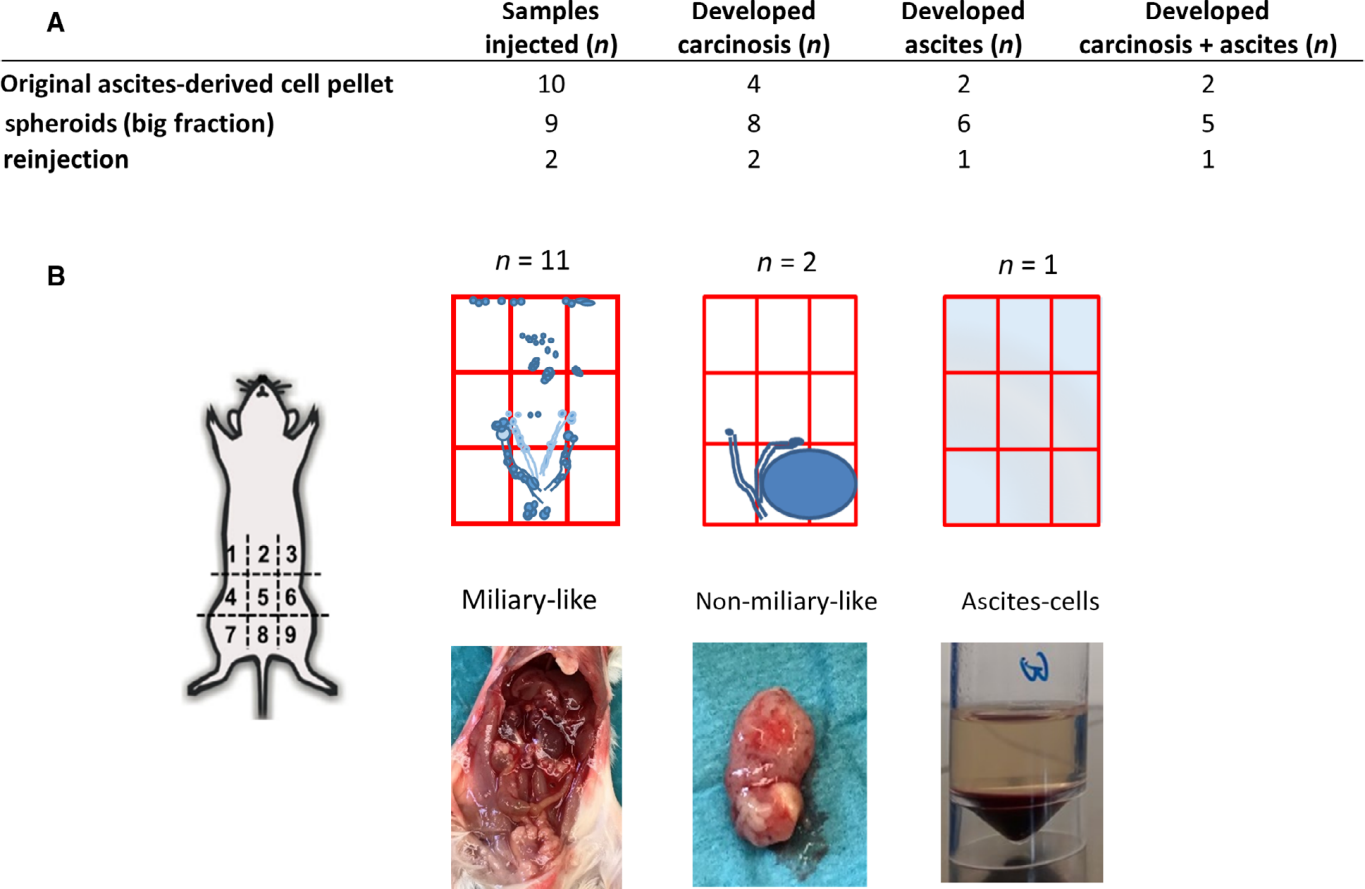
*In vivo* behavior of ascites‐derived cells. (A) Number of ascites‐derived samples analyzed *in vivo*: I.p. injection of original ascites cell pellets (*n* = 10) led to tumor formation in four cases and in two of them additionally to ascites formation. All spheroid samples (big fraction, *n* = 9) developed metastatic ovarian cancer. All reinjected samples developed peritoneal carcinosis as well. (B) Three main dissemination patterns were observed, a miliary‐like, a non‐miliary like and solely ascites. The metastatic spread pattern in the mice resembled the tumor dissemination pattern in the corresponding patient.

In a next step, we were interested in the tumorigenicity of the different populations found in the ascites‐derived cells *in vivo*. Therefore, ADs, spheroids (cell fraction size > 15 μm), single cells (cell fraction size < 15 μm), and/or original pellets from different high‐grade serous ovarian cancer patients (*n* = 21) were each intraperitoneally injected in immunodeficient mice. None of the mice injected with ADs (*n* = 7) developed peritoneal carcinomatosis, whereas injection of the original ascites cell pellets, without cell separation or previous cultivation, led to tumor or ascites formation in 50% and 20% of the injected samples, respectively. Similarly, all spheroid samples (*n* = 9) gained from the original pellet via filtration with a cell strainer developed in a time period ranging from 18 to 56 weeks metastatic ovarian cancer, thereby showing different extents of dissemination and patterns (Fig. 4). For two cases, the corresponding single‐cell fraction was injected in parallel. Here, no tumor formation could be observed, even though this fraction contains single tumor cells, as showed before. Interestingly, we found a strong similarity in the tumor dissemination pattern observed in mice and in the corresponding ovarian cancer patient, as described in the surgery protocol. In our mouse model, we could clearly distinguish between a miliary‐like dissemination pattern found in 11 samples and a ‘non‐miliary’ tumor spread generated by 2 samples, the last characterized by one or two large tumor bulks within the peritoneal cavity or retroperitoneal located (*n* = 2). One sample developed only malignant ascites, containing large amounts of tumor cell spheroids without any solid tumor lesion. Remarkably, reinjection of mouse ascites‐derived tumor cells led to tumor and to some extent to ascites development in all cases (*n* = 2), thereby maintaining the same spread pattern and showing faster progression rates.

### 3.4 Transcriptome of tumor cell spheroids from HGSOC patients

**Fig. 5:**
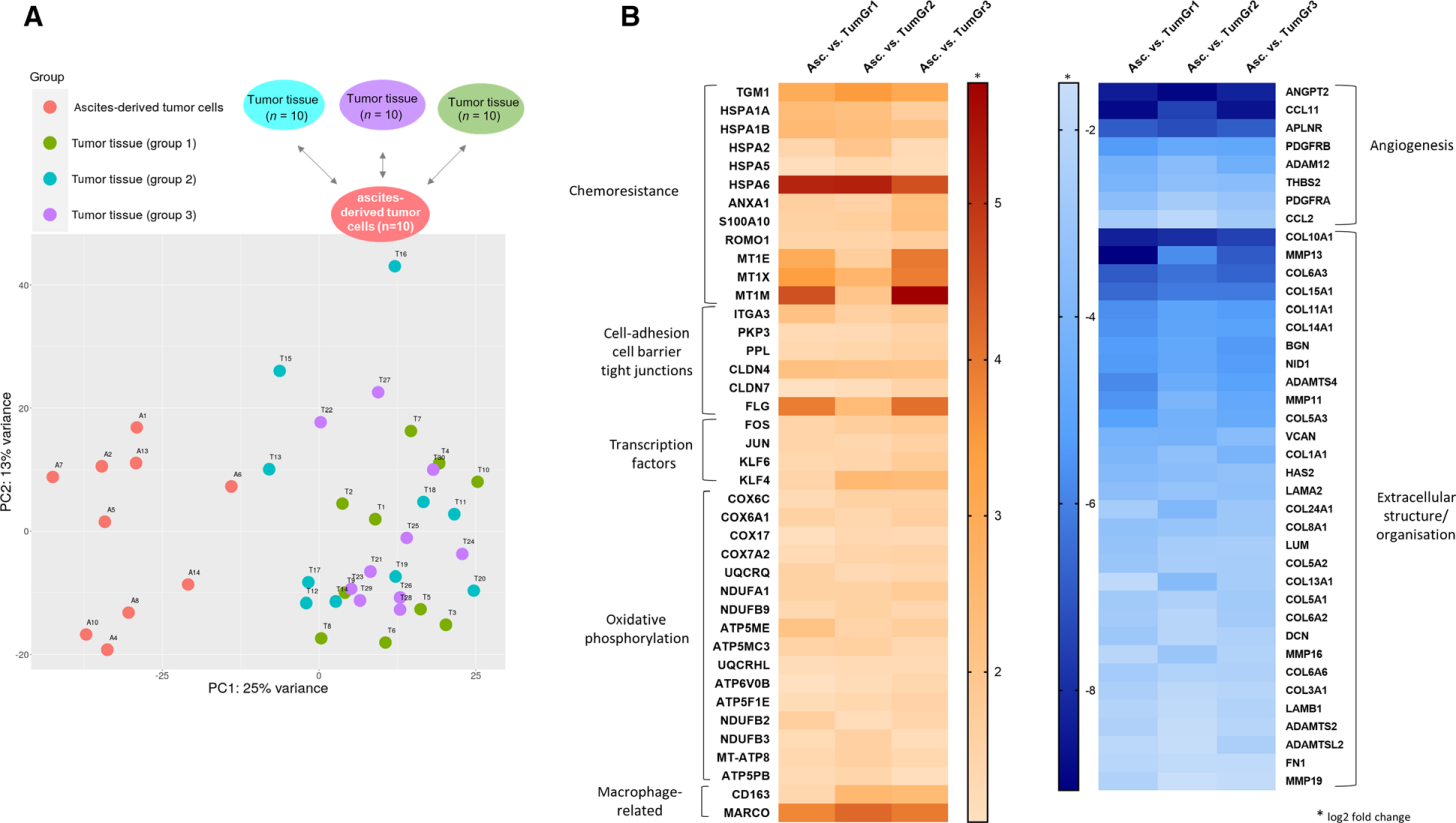
RNA‐seq analysis of ascites‐derived spheroids. (A) PCA Plot showing a clear separation or the ascites‐spheroid samples (A1–A10) and the tumor sample group (T1–T30). (B) Hit maps displaying selected up‐regulated and down‐regulated genes in the spheroid tumor cells in comparison with the tumor cells from primary or metastatic tissue samples.

As shown in the *in vivo* model, ascites‐derived tumor cell spheroids from ovarian cancer patients can be considered ‘metastatic units’ that promote peritoneal carcinomatosis as well as retroperitoneal tumor cell dissemination. We assume that tumor cell spheroids biologically differ from those tumor cells in the primary or metastatic tumor tissue, since they must be able to survive as floating units and eventually to attach at the metastatic site. In order to identify the molecular players responsible for these specific biological characteristics, the transcriptome of ascites‐derived tumor spheroids and tumor tissue samples were compared. RNA‐seq analysis from 10 different tumor spheroid patient samples and 30 tumor tissue samples, including primary and metastatic tissue, was performed. Here, the ascites‐spheroid (A1–A10) and the tumor sample group (T1–T30) form two distinct clusters in the principal component analysis (PCA; Fig. 5A). Subsequently, comparative expression analyses between the ascites group and three different tumor groups, each one containing 10 tumor samples, were carried out (Fig. 5B). The tumor tissue samples included in each of the three groups were selected based on the patient characteristics (FIGO, age, histology, grading, tumor stage, and lymph node status) to resemble the ascites group. Only those genes commonly and significantly (type="InBasic_Latin">|Log_2_‐fold change| > 1 and FDR < 0.1) de‐regulated in the ascites‐derived spheroids in comparison with all three tumor groups were further evaluated and are displayed in the Tables S2 and S3. A selected group of these genes has been presented in the heat map on Fig. 5. Interestingly, the oxidative phosphorylation pathway, including a large number of genes, that is, ATP synthases, NADH oxidoreductases, and cytochrome c oxidases, are significantly up‐regulated (*P* value: 5.6952e^−12^, FDR: 1.6539e^−8^) in the ascites spheroids in comparison with the tumor cells from the primary or metastatic tissue. Higher mRNA levels of genes related to chemoresistance, that is, (*TGM1*) transglutaminase 1, heat shock proteins and metallothioneins, to cell–cell adhesion and also barrier molecules, that is, (*PKP3*) plakophilin 3, (*PPL*) periplakin, (CLDN4/7) claudin 4/7, and (*FLG*) filaggrin, were found in ascites spheroids versus tumor tissue as well. Three glycosylation enzymes, *NEU1* (sialidase 1), (*B4GALT5*) beta‐1,4‐galactosyltransferase 5, and (*CHST4*) carbohydrate sulfotransferase 4 as well as several transcription factors, (*FOS*) c‐fos, (*JUN*) c‐jun and (KLF4/6) kruppel‐like factor 4/6 were also up‐regulated in the ascites group. Interestingly, the increased expression of *CD163* and (*MARCO*) macrophage receptor with collagenous structure in the group of ascites‐derived spheroids suggests an important role of macrophages on the biology of these cellular structures. Among the 1316 significantly down‐regulated genes in the ascites spheroids, two main pathways: angiogenesis and extracellular structure organization could be identified, which are significantly down‐regulated (*P* value and FDR are almost 0). Numerous genes involved in angiogenesis, that is, (*ANGTP2*) angiopoietin‐2, (*APLNR*) apelin receptor, (*PDGFRβ*) platelet‐derived growth factor receptor beta or the cytokines (*CCL11*) C‐C motif chemokine 11 and (*CCL2*) chemokine (C‐C motif) ligand 2, and as expected in the extracellular structure organization (i.e., several collagen proteins, matrix metalloproteinases MMP16/19, fibronectin, lumican, versican, and the hyaluronan synthase 2).

### 3.5 Mitochondrial function and OXPHOS inhibition in tumor spheroids from HGSOC patients

**Fig. 6:**
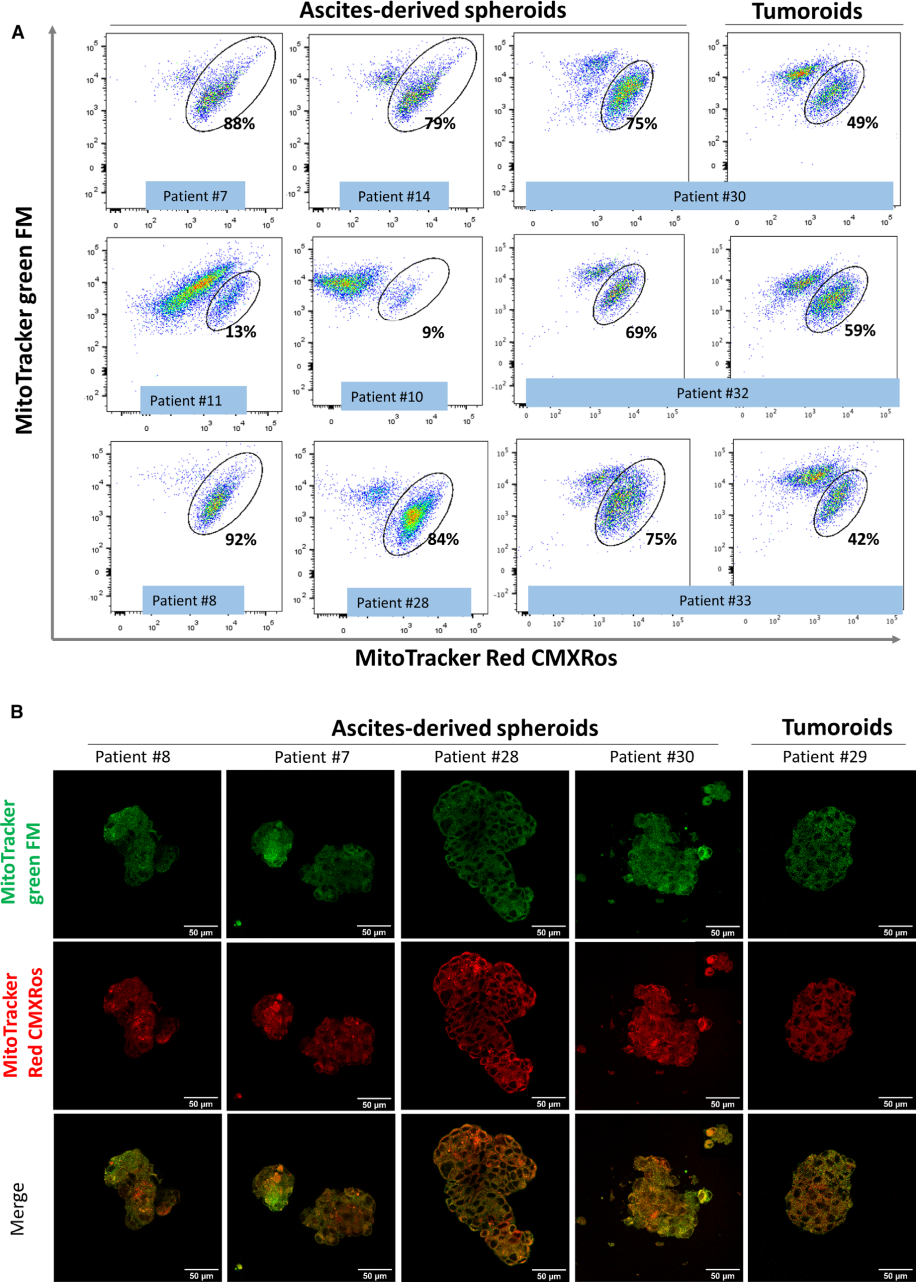
Staining with mitochondrial markers. Ascites‐derived tumor spheroids and tumoroids from tumor tissue were stained with markers for mitochondrial mass and membrane potential using MitoTracker green FM and MitoTracker Red CMXRos, respectively. (A) Evaluation using flow cytometry showed high mitochondria mass levels in all samples and two subpopulations with different mitochondrial membrane potential. The subpopulation showing a higher mitochondrial membrane potential has been quantified in all samples including three matched pairs. (B) Confocal microscopy analyses (60×) ascites‐derived tumor spheroids and tumoroids from tumor tissue stained with MitoTracker green FM and MitoTracker Red CMXRos.

We further focused our attention on the role of OXPHOS upregulation and subsequently analyzed markers for mitochondrial mass and membrane potential using MitoTracker green FM and MitoTracker Red CMXRos, respectively, in ascites‐derived tumor spheroids and tumor tissue from HGSOC patients. MitoTracker Red CMXRos can be used to determine mitochondrial functionality and a mitochondrial membrane potential loss has been associated with the metabolic shift from OXPHOS to glycolysis [14, 15]. In FACS analysis, all samples independently of their origin showed high mitochondria mass levels using MitoTracker green FM, whereas two subpopulations with different mitochondrial membrane potential were visible after MitoTracker Red CMXRos staining using flow cytometry. Among the nine spheroid samples, seven showed a large population (≥ 75%) with high membrane potential and only two spheroid samples showed lower values (9% and 13%). For three ascites‐derived spheroids, the corresponding tissue material was available. Here, we observed a reduced subpopulation of cells with higher membrane potential levels in the tissue samples, namely 49%, 59%, and 42% compared with the matched spheroid probes showing 75%, 69%, and 75%, respectively (Fig. 6A). In the fluorescence microscopy, we evaluated the staining pattern of the mitochondrial markers in 4 ascites‐derived tumor spheroid and one tumor tissue sample. As shown exemplary in Fig. 6B, we observed a homogeneous staining for both markers (mitochondrial mass and membrane potential) in the tumoroid structures, whereas in the tumor spheroids from ascites, a stronger MitoTracker Red CMXRos staining was detected in certain areas, suggesting the presence of a tumor subpopulation with higher mitochondrial activity.

**Fig. 7:**
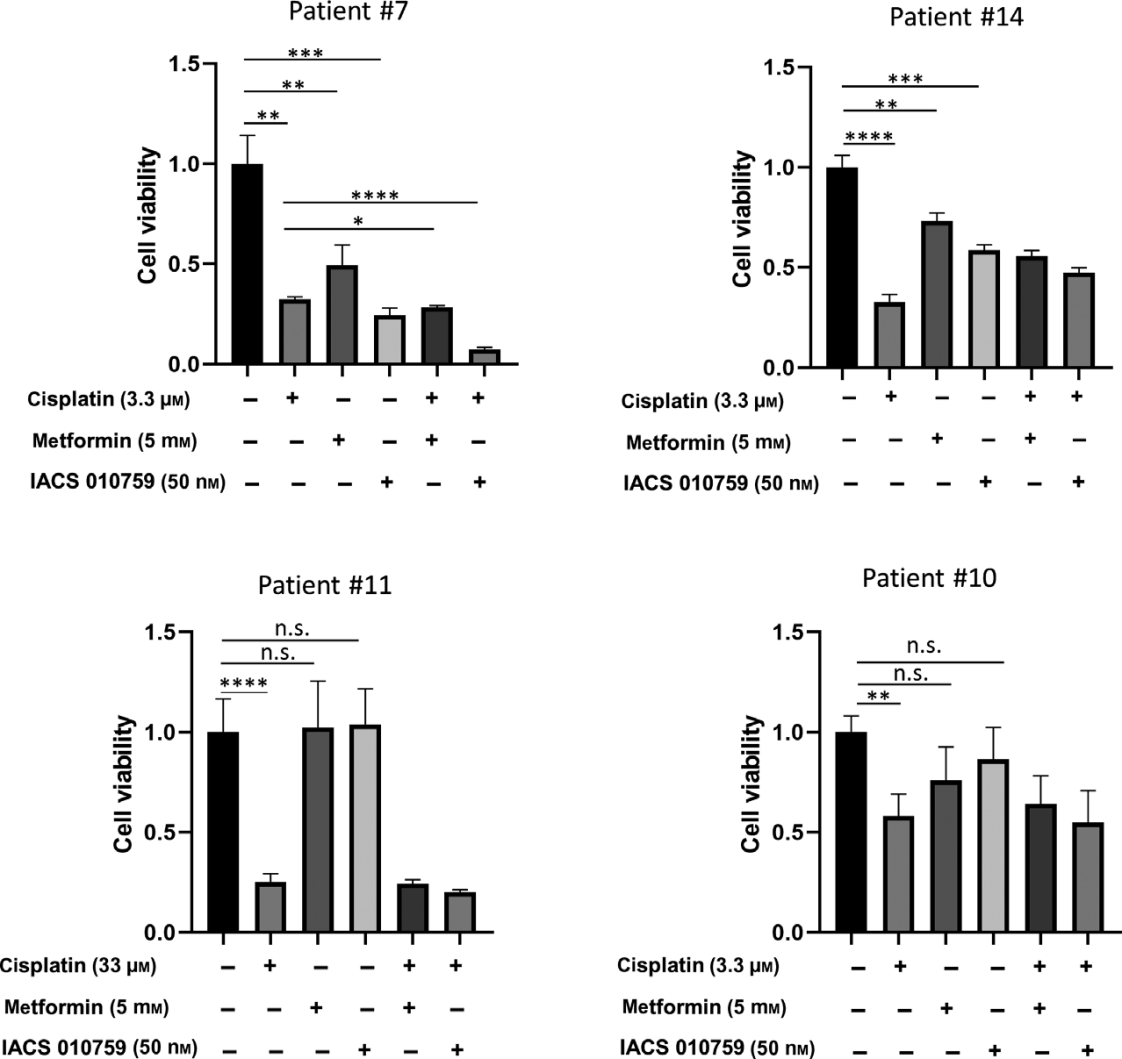
Inhibition of the OXPHOS pathway via metformin and IACS 010759 treatment in tumor spheroids from HGSOC patients. Four samples from different patients were incubated with increasing metformin (5 mm), IACS 010759 (50 nm), and cisplatin (3.3 and 33.3 µm) for 48 h and cell viability was subsequently assessed. Two samples showed a strong response to the metformin treatment and simultaneous treatment with metformin and cisplatin led to a significant viability reduction when compared with each treatment alone. Samples #10 and #11 showed no response to OXPHOS inhibition, whereas they were highly sensitive to cisplatin treatment. All the results were shown as mean ± SD (*n* = 3). Statistical significance was determined using unpaired two‐tailed Student’s *t*‐tests; *P < 0.05, **P < 0.01, ***P < 0.001 and ****P < 0.0001.

In order to evaluate the effect of an OXPHOS pathway inhibition on ascites‐derived tumor spheroids from HGSOC patients, *in vitro* cell viability was measured after treatment with two OXPHOS inhibitors, metformin and IACS 010759, either alone or in combination with cisplatin. Here, four samples from different patients were incubated with metformin (5 mm), IACS 010759 (50 nm) and cisplatin concentrations (3.3 and 33.3 µm) for 48 h. Cell viability was assessed subsequently using CellTiter‐Glo solution as described in the methods section. Among the four samples analyzed, two samples (#7 and #14) showed a strong response to OXPHOS inhibition and in one of them the simultaneous treatment with OXPHOS inhibitors and cisplatin led to a significant viability reduction when compared with each treatment alone. Two samples (#11 and #10) showed no response to metformin or IACS 010759 as single treatment, whereas there were sensitive to cisplatin treatment (Fig. 7). Interestingly, these no‐responder #10 and #11 displayed in the FACs analysis low mitochondrial membrane potential levels measured by MitoTracker Red CMXRos staining as shown in Fig. 6A.

## 4 Discussion

In ovarian cancer, detached single and tumor cell aggregates (spheroids) from the primary tumor that persist in the peritoneal fluid represent the main source of intraperitoneal metastasis [16]. Specially, tumor cells within the spheroids exhibit a survival benefit and may represent a key element of chemotherapy‐sensitive recurrence. In the present study, we were able to identify and enrich this tumorigenic subpopulation within the ascites of ovarian cancer patients and further elucidate via RNA‐seq analysis unique molecular characteristics of these cellular structures. Here, we have focused our attention at the high‐grade serous ovarian cancer, since it represents the most frequent subtype.

Malignant ascites itself constitutes a favorable milieu for tumor cells to progress. It contains soluble factors such as cytokines, chemokines, growth factors, and extracellular matrix fragments as well as a complex mixture of cells including tumor, stromal cells, and infiltrating immune cells [2]. The cellular part of ascites includes single cells and cell aggregates, so‐called floating spheroids [5, 6]. In the present study, we could show that the single‐cell population includes some tumor cells, but it is principally composed of immune cells and to a less extent of a mesenchymal‐like cell population, defined in our analysis by a CD90 positivity. In contrast, the cell aggregates found in most ovarian cancer samples contained a much higher (> 80%) percentage of tumor cells showing a strong EpCAM expression or combined EpCAM and CD24 positivity. Here, the tumor purity as well as the protective environment created by these structures may explain the high rate of successful tumor development observed, when injecting tumor spheroids intraperitoneally in immunodeficient mice, in contrast with single cell tumor injections. Moreover, the interaction between tumor cells and other cellular components within the spheroids seems to be essential in order to keep their compact structure, but it also enhances the survival ability and invasive potential of the tumor cells. In this context, different cell populations such as cancer‐associated fibroblasts (CAFs) or tumor‐associated macrophages (TAMs) have been described as key players for the aggregation as well as for the adhesive and invasive properties of these tumor cell structures, thereby potentiating their malignant phenotype and facilitating the peritoneal metastatic process [17, 18, 19, 20]. Our FACs and RNA‐seq analysis on purified tumor spheroids are in line with these findings. We could frequently detect CD45‐positive and CD90‐positive cells within the spheroids, although both represented a small fraction within the spheroids, and further two macrophage‐associated genes (CD163 and MARCO) were found to be highly up‐regulated in the tumor spheroids in comparison with the solid tumor tissue. CD163 is a characteristic marker of M2 macrophages, which are the most predominantly TAM subtype found in ovarian cancer and are associated with tumor invasion, angiogenesis, metastatic disease, and early recurrence [20, 21, 22]. MARCO is a class A scavenger receptor expressed by immune‐suppressive tumor‐associated macrophages and has been linked to poor prognosis in breast cancer [23, 24]. Interestingly, targeting MARCO‐positive TAMs with a specific antibody reduces tumor growth and metastasis in breast, colon, and melanoma mouse models [25]. In glioblastoma, MARCO‐expressing TAMs induce a phenotypic shift toward mesenchymal cellular state of glioma stem cells, promoting both invasive and proliferative activities, as well as therapeutic resistance to irradiation [26]. Additional analyses are required in order to elucidate the impact of MARCO‐positive TAMs on spheroid tumor cells and whether this interaction might further influence disease progression of ovarian cancer patients.

One characteristic feature of tumor spheroids is their low chemosensitivity, in part attributed to a low proliferative profile [3]. The standard chemotherapy for ovarian cancer patients, consisting in a paclitaxel and carboplatin combination, selectively targets and eliminates highly proliferative tumor cells [27]. In poorly vascularized tumor areas, however, cells become quiescent [28] and in turn less responsive to therapy. A recent study has estimated that in ovarian cancer spheroids more than 60% of the cells are quiescent [4]. Moreover, it has been described that quiescent tumor cells use preferentially the mitochondrial OXPHOS pathway for their ATP production [29].

In line with this data, our RNA‐seq analysis revealed a significant upregulation of the OXPHOS pathway in the tumor spheroids isolated from the ascites of ovarian cancer patients compared to corresponding solid tumor tissue samples. In this context, we assume that the metabolic switch confers ascites‐derived tumor spheroids a survival benefit and in turn contributes to an increased metastatic potential. Thus, OXPHOS pathway inhibition opens an attractive therapeutic window for the specific target of tumor spheroids, as the major vehicle of peritoneal metastasis in OvCa. By using OXPHOS inhibitors, spheroid tumor cells might not be able to cover their high ATP demand. Contrary to normal cells that can activate glycolysis in response to OXPHOS inhibition, quiescent tumor cells within the spheroids have no access to sufficient glucose in order to compensate the loss of ATP production and might die [30]. Interestingly, several drugs, including metformin, that have been used clinically for non‐oncologic indications have emerged as effective OXPHOS inhibitors [31]. Several cohort studies have described a protective effect and an association of metformin with longer overall survival in ovarian cancer patients [32, 33, 34, 35]. Also, two *in vitro* studies have found reduced ovarian cancer cell proliferation, migration, and increased apoptosis [36] as well improved sensitivity in drug‐resistant ovarian cancer cell lines [37] after metformin treatment. In contrast, a recent meta‐analysis that excluded studies considered to have the potential for immortal time bias suggested no overall survival benefit associated with use of metformin [38]. A recent pilot study evaluating the efficacy of metformin plus first‐line chemotherapy versus chemotherapy alone in a small cohort of ovarian cancer patients found no effects of metformin, neither[39] Our *in vitro* analyses might explain these contradictory findings. Here, ascites‐derived tumor spheroids from different HGSOC patients showed response to metformin or to the OXPHOS inhibitor IACS 010759 only to some extent, indicating that inhibition of the OXPHOS pathway might be not a universal target for HGSOC patients. Interestingly, our analyses showed that the level of mitochondrial membrane potential in the ascites‐derived tumor spheroids strongly correlates with OXPHOS inhibition treatment response *in vitro*. Thus, the quantification of the mitochondrial function in ascites‐derived cells might represent an attractive tool to discriminate between responders and non‐responders in terms of an OXPHOS therapy.

Our RNA‐seq data have further revealed several factors up‐regulated in spheroids that are linked to cell chemoresistance. The transglutaminase 1 (TGM1), an enzyme that is mainly found in the epidermis, catalyzes protein bonds, so‐called cross‐linking, which give the tissue strength and stability. In gastric carcinoma TGM1 has been shown to promote the stem cell character and chemoresistance of tumor cells via modulation of the Wnt/beta‐catenin signaling pathway. Further, several members of the heat shock protein 70 family (Hsp70) were found to be significantly up‐regulated in the tumor spheroids compared with the tumor tissue. The human Hsp70 family consists of eight highly homologous members of chaperone molecules that differ in their intracellular localization and expression pattern. Specially, *HSPA1A/1B* and *HSPA6*, which code for the proteins Hsp70 and Hsp70‐6, respectively, are only expressed at low or undetectable levels under physiological conditions, but are rapidly induced by cellular stress [40]. In cancer cells, the effect of Hsp70 has been not only related to its chaperone activity, but rather to its antiapoptotic role and the regulation of cell signaling. In ovarian cancer, increased Hsp70 expression was found in chemoresistant cells. Here, Hsp70 proteins block the translocation of Bax into the mitochondria and the release of mitochondrial proteins into the cytosol [41]. Additionally, three metallothioneins (*MT1E*, *MT1M,* and *MT1X*) were highly up‐regulated in the spheroids. MTs are small cysteine‐rich proteins with a key role in metal homeostasis and protection against heavy metal toxicity. Consequently, a drug resistance function has been described in the context of cancer [42], though specifically in ovarian cancer no difference between MT expression in tumors from chemotherapy‐treated versus untreated patients could be found [43]. Still, MT expression has been negatively associated with survival time in primary ovarian carcinomas [44].

Besides the low proliferative rate and chemosensitivity, tumor cells within the spheroids might acquire specific adhesive characteristics that support a protective and compact cellular aggregation structure [45]. In this context, our RNA‐seq analysis revealed high mRNA levels of integrin α3 (*ITGA3*), claudins 4 and 7 (CLDN4/7), desmosome proteins plakophilin 3 (*PKP3*) and periplakin (*PPL*) as well as the barrier protein filaggrin (*FLG*), the last showing an aprox. 10‐fold upregulation in the ascites‐derived spheroids compared with tumor tissue. Interestingly, recent data raised the possibility that molecules with mechanical barrier function may be used by cancer cells to protect them from immune cell infiltration and immune‐mediated destruction. Here, authors identified eight genes, including *PPL* and *PKP3*, whose increase expression in human melanoma metastases and ovarian cancers was associated with a lack of Th1 immune signatures and further strongly correlated with shorter overall survival [46].

The *in vitro* and *in vivo* behavior of ascites‐derived cells has been reported by other groups before [2, 45, 47, 48]. In our study, we showed similar results as previously described, namely in the majority of the samples ascites tumor spheroids from ovarian cancer patients showed a quiescent and non‐adhesive phenotype when cultured *in vitro*, whereas the single cells gave rise to an adherent and highly proliferative population. In contrast, intraperitoneal injection in immunodeficient mice showed just the opposite picture; namely, tumor development was observed in most of the spheroid samples, but none of the ADs developed carcinosis. FACS and ICC analyses revealed an explanation for this contrary behavior, showing that the spheroids consist principally of tumor cells population, whereas the single cells were mainly of non‐epithelial origin. We assume the lack of an adequate stimulus *in vitro* prevent tumor spheroids to attach and further spread, thereby highlighting the key role of the intraperitoneal environment for tumor progression in ovarian cancer. Thus, the key role of fibroblasts, immune, adipocytes, mesothelial, and endothelial cells for disease progression has been broadly described in the last years [49, 50].

Remarkably, two samples showed a totally different pattern regarding their cellular distribution and *in vitro* behavior. Here, the AD populations included a high percentage of tumor cells, as they showed a strong staining for EpCAM. These results emphasize the high heterogeneity of ‘ovarian cancer’ and the need to decipher the different biological subtypes behind this entity, in order to develop specific and targeted therapies. In this context, the ascites‐derived tumor spheroids might represent a suitable model to address this question, especially if we consider that their dissemination pattern in the mice clearly mimic the one observed in the patient.

## 5 Conclusion

In the present study, we could show that ascites‐derived spheroids from high‐grade serous ovarian cancer patients are highly tumorigenic *in vivo* and clearly depict the biology and metastatic pattern of the individual disease. Moreover, we could identify by transcriptome analysis several molecular markers involved in chemoresistance (*TGM1*, HSPAs, MT1s), cell‐adhesion and cell barrier (*PKP3*, CLDNs, *PPL*) that might help us to better understand the special characteristics of ascites‐derived tumor spheroids. Specially, the upregulation of the OXPHOS pathway suggests a metabolic switch in the ascites‐derived tumor spheroids compared to primary or metastatic tissue. Here, OXPHOS inhibition using metformin led to a strong viability reduction in tumor spheroids from different HGSOC patients *in vitro*. Ongoing analyses in our group aim to decipher the mechanisms of mitochondria metabolism activation and to corroborate the therapeutic efficacy of OXPHOS inhibition in a personalized manner.

## Conflict of interest

The authors declare no conflict of interest.

### Peer Review

The peer review history for this article is available at https://publons.com/publon/10.1002/1878‐0261.13028.

## Data accessibility

All data generated or analyzed during this study are included in this published article and its supplementary information files.

## Author contributions

YD performed the experiments, analyzed data, and contributed to write the manuscript. VL contributed to the animal experiments. KL contributed to the production of the RNA‐Seq data. MYQ analyzed the RNA‐Seq data. BS provided patient material and characteristics. CS and US designed the mouse experiments. LOF conceptualized the project, designed the experiments, and was a major contributor in writing the manuscript. All authors read, reviewed, and approved the final manuscript.

## Consent for publication

Not applicable.

## Supporting information


**Fig. S1**. FACs analysis of additional ADs and NADs cells from ascites samples.

**Fig S2**. Two AD samples showing a different behavior *in* *vitro*. They both contained low proliferative AD cells with an additional strong EpCAM, CD24, CD133 staining, suggesting a cancer stem cell phenotype.

**Table S1**. Patient characteristics.

**Table S2**. Up‐regulated genes in ascites‐derived spheroids compared with tumor tissue.

**Table S3**. Down‐regulated genes in ascites‐derived spheroids compared to tumor tissue.
