# Supplementary figures and images for: Molecular characteristics and tumorigenicity of ascites‐derived tumor cells: mitochondrial oxidative phosphorylation as a novel therapy target in ovarian cancer
Source: Mol Oncol. 2021 Jun 18;15(12):3578–95. doi: 10.1002/1878-0261.13028 (PMC8637562; doi:10.1002/1878-0261.13028)

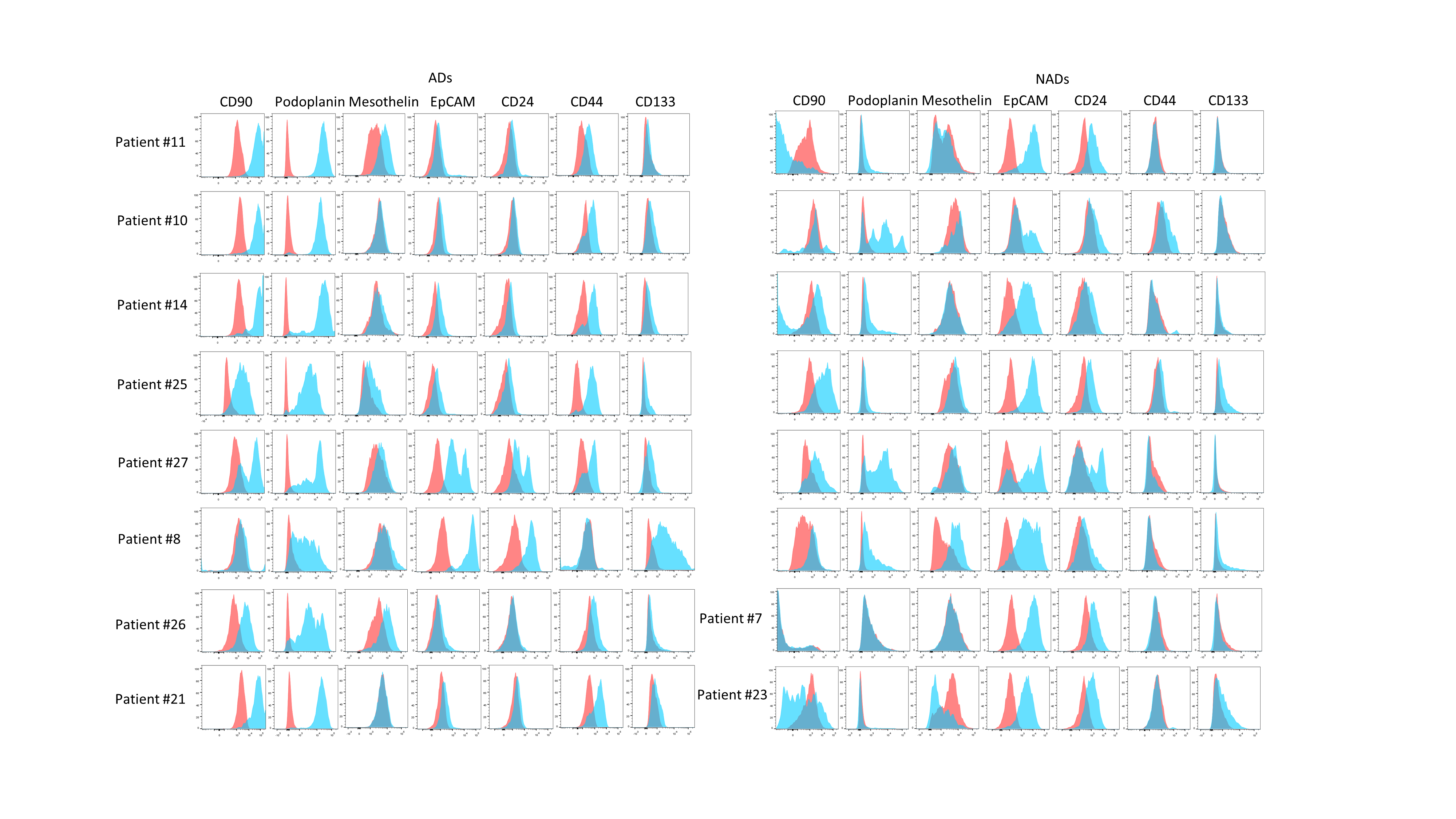

Supplement: Supplementary file 1 — Fig. S1. FACs analysis of additional ADs and NADs cells from ascites samples. [file MOL2-15-3578-s002.tif]

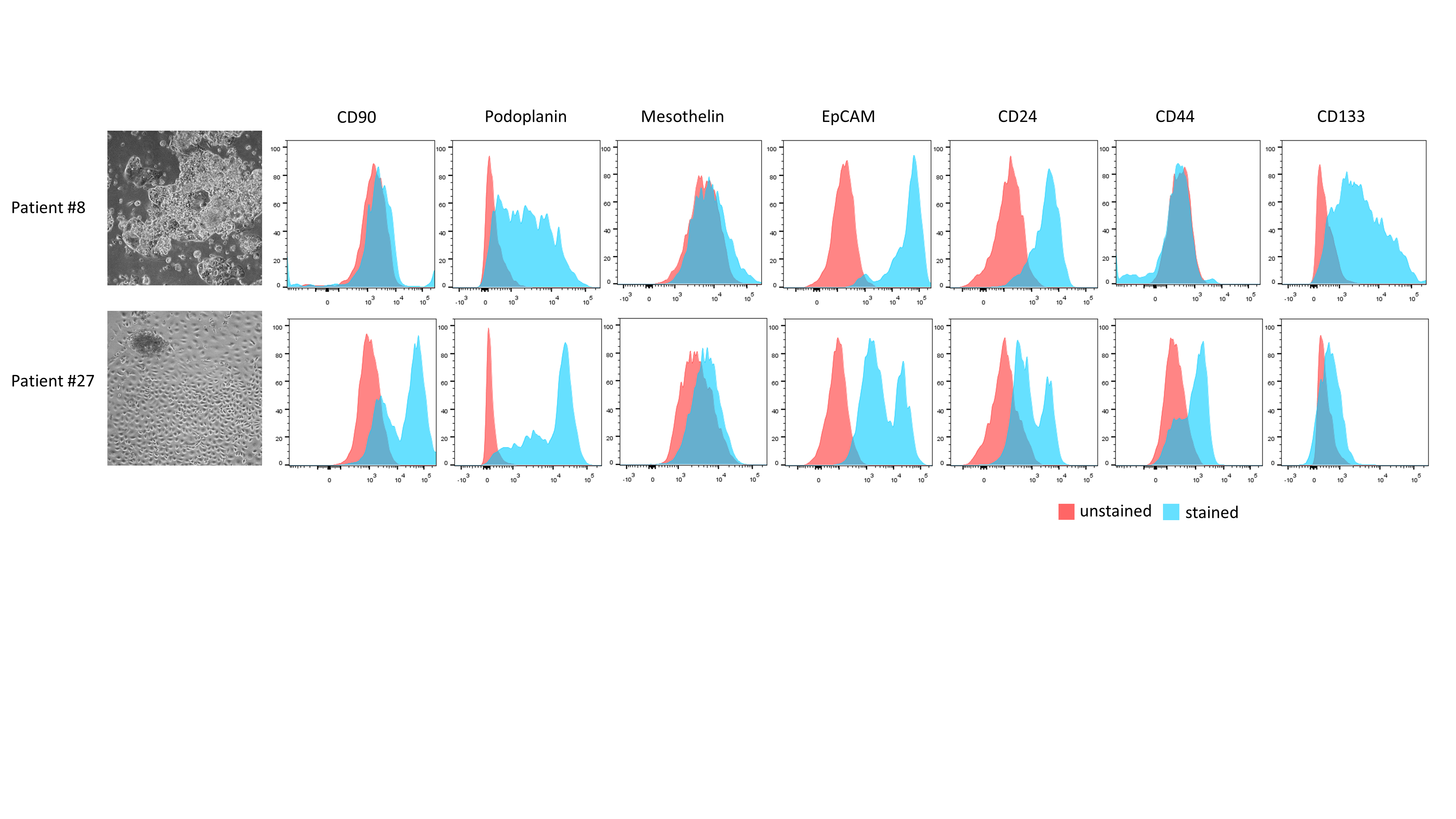

Supplement: Supplementary file 2 — Fig S2. Two AD samples showing a different behavior in vitro. They both contained low proliferative AD cells with an additional strong EpCAM, CD24, CD133 staining, suggesting a cancer stem cell phenotype. [file MOL2-15-3578-s004.tif]
